# Severe-combined immunodeficient rats can be used to generate a model of perinatal hypoxic-ischemic brain injury to facilitate studies of engrafted human neural stem cells

**DOI:** 10.1371/journal.pone.0208105

**Published:** 2018-11-28

**Authors:** Stephanie R. Beldick, James Hong, Svetlana Altamentova, Mohamad Khazaei, Anisha Hundal, Mohammad-Masoud Zavvarian, Prakasham Rumajogee, Jonathon Chio, Michael G. Fehlings

**Affiliations:** 1 Institute of Medical Science, University of Toronto, Toronto, Ontario, Canada; 2 Division of Genetics and Development, Krembil Research Institute, Toronto, Ontario, Canada; 3 Life Sciences Program, University of Toronto Mississauga, Mississauga, Ontario, Canada; 4 Division of Neurosurgery, University of Toronto, Toronto, Ontario, Canada; University of Modena and Reggio Emilia, ITALY

## Abstract

Cerebral palsy (CP) encompasses a group of non-progressive brain disorders that are often acquired through perinatal hypoxic-ischemic (HI) brain injury. Injury leads to a cascade of cell death events, resulting in lifetime motor and cognitive deficits. There are currently no treatments that can repair the resulting brain damage and improve functional outcomes. To date, preclinical research using neural precursor cell (NPC) transplantation as a therapy for HI brain injury has shown promise. To translate this treatment to the clinic, it is essential that human-derived NPCs also be tested in animal models, however, a major limitation is the high risk of xenograft rejection. A solution is to transplant the cells into immune-deficient rodents, but there are currently no models of HI brain injury established in such a cohort of animals. Here, we demonstrate that a model of HI brain injury can be generated in immune-deficient *Prkdc* knockout (KO) rats. Long-term deficits in sensorimotor function were similar between KO and wildtype (WT) rats. Interestingly, some aspects of the injury were more severe in KO rats. Additionally, human induced pluripotent stem cell derived (hiPSC)-NPCs had higher survival at 10 weeks post-transplant in KO rats when compared to their WT counterparts. This work establishes a reliable model of neonatal HI brain injury in *Prkdc* KO rats that will allow for future transplantation, survival, and long-term evaluation of the safety and efficacy of hiPSC-NPCs for neonatal brain damage. This model will enable critical preclinical translational research using human NPCs.

## Introduction

Hypoxic-ischemic (HI) brain injury is a major cause of neurological dysfunction in neonates [1,2]. Injury to the brain during the perinatal time period often leads to a diagnosis of cerebral palsy (CP) when children fail to meet developmental milestones. The incidence of CP is approximately 2.5/1000 live births, with individuals displaying a wide range of motor and cognitive deficits that cause lifelong disability [1,3]. The financial burden of CP is significant, with lifetime healthcare costs estimated at approximately 900,000 USD per patient [1]. There is therefore a need to develop therapies that can improve the quality of life for these patients as well as reduce the economic impact on society.

In 1981, Rice and Vannucci developed what is currently the most commonly used preclinical model of neonatal HI brain injury. The Rice-Vannucci model is an adaptation of the Levine model, consisting of common carotid artery occlusion and systemic hypoxia that leads to unilateral brain damage [2]. Applying this model at postnatal day (P)7 in rats generates an injury in both white and grey matter that recapitulates injuries seen in term neonates [4,5]. Many parameters of the injury model can be modified to suit the needs of the experimental question. Time of surgery, length of post-operative recovery, length and extent of hypoxic conditions, as well as temperature all have an impact on the outcome of the injury [6–8].

HI brain injury produces a cascade of destructive mechanisms that result in necrosis and apoptosis of neurons and oligodendrocytes. When cells of the brain experience hypoxic conditions, the homeostatic balance of the cell is disrupted, leading to mitochondrial dysfunction and subsequent ATP depletion, glutamate excitotoxicity via the over-activation of *N*-methyl-D-aspartate (NMDA) receptors, and generation of reactive oxygen species [9,10]. These processes lead to cell death accompanied by the production of astrogliotic scarring, activation of local microglia, and infiltration of circulating immune cells [9,10].

Presently there are no available treatments that can repair the brain damage caused by an HI insult. Hypothermia is currently applied as a neuroprotective treatment at birth for term infants at risk of developing neurological deficits, however, approximately half of the infants treated still go on to develop neurological impairments [11]. As a consequence, it is essential that other therapies be developed to reduce the extent of brain damage and functional deficits.

Transplantation of neural precursor cells (NPCs) into the brains of patients who have experienced HI injuries has the potential to fill this gap in therapies. NPCs have the advantage of being inherently predisposed to differentiate into all cell types found in the central nervous system (CNS). Compared with other stem cell types found in umbilical cord blood or bone marrow, this preferential differentiation positions NPCs to have the greatest potential to integrate into the CNS environment [3,12]. Despite their potential, NPCs have only been assessed to a moderate extent in preclinical models and have had limited testing in the clinic. Preclinical studies performing transplantations of rodent NPCs into the brains of injured animals have shown behavioural and anatomical recovery [13–16]. NPCs are thought to work through various mechanisms, including replacing damaged cells and mediating endogenous cell repair mechanisms through the release of trophic factors [3,17,18].

To promote translation of NPC transplantation to the clinic, it is essential that the safety and efficacy of human-derived NPCs first be evaluated in preclinical models. While some studies have worked toward this goal [19–22], xenograft rejection, which occurs in immune-competent animals, is a major blockade to studying the long-term effects of human-derived NPCs on brain injury [23]. In order to circumvent this hurdle, transplantation of human-derived NPCs into immune-deficient animals is an appealing approach [23,24].

*Prkdc* knockout (KO) rats are a strain of severe-combined immunodeficient (SCID) rats that were developed by Mashimo et al. in 2012 [25]. Using zinc finger nuclease technology, the researchers created a loss of function mutation in the first exon of the *Prkdc* gene. This gene is an essential component of the non-homologous end joining process in V(D)J recombination, which is necessary for the formation of T and B cell receptors [25]. A lack of these receptors prevents the selection and survival of T and B cells during their development in the thymus and bone marrow, and consequently, *Prkdc* KO rats are lacking in both these cell types. T and B cells play major roles in xenograft rejection after transplantation through both humoral and cellular immune responses [26].

Therefore, utilizing *Prkdc* KO rats to generate a model of neonatal HI brain injury will allow for long-term studies of human-derived NPC transplantation as a treatment for neonatal stroke and CP. Importantly, it is well established that the species and strain of animals has an effect on HI brain injury generation [27]. Therefore, it is essential for this model to be well-characterized in *Prkdc* KO rats in order to provide a reliable platform through which human stem cells can be tested. After characterization, human-derived NPC survival must be evaluated in both *Prkdc* KO and wildtype (WT) HI animals to confirm the utility of this model for future treatment studies.

In the present study, we aimed to evaluate the feasibility of generating a modified version of the Rice-Vannucci HI brain injury model in SCID rats. We assessed the injury from a (1) phenotypic perspective using sensorimotor behavioural tests and (2) through histological analyses of the brain. Patients diagnosed with CP due to perinatal asphyxia most commonly present with motor difficulties, and thus we sought to evaluate sensorimotor impairments in this rodent model. Histological analysis is a widely accepted, reliable, and relatively inexpensive approach to assessing changes in the brain following injury. After establishing the baseline HI model in SCID rats, we proceeded to transplant human induced pluripotent stem cell (hiPSC)-NPCs into the injured brain. hiPSC-NPCs were chosen for their high clinical potential to circumvent allogeneic rejection and ethical sourcing concerns [28]. Since the focus of this work was aimed at establishing a robust baseline model for future xenograft studies, we assessed cell survival without diving deeper into evaluating the cells’ effects on recovery, which will certainly be the next step. All experiments were compared to immune-competent WT littermates in order to evaluate any effect of genotype on the injury generation and cell survival.

## Materials and methods

### Animals

**Fig 1:**
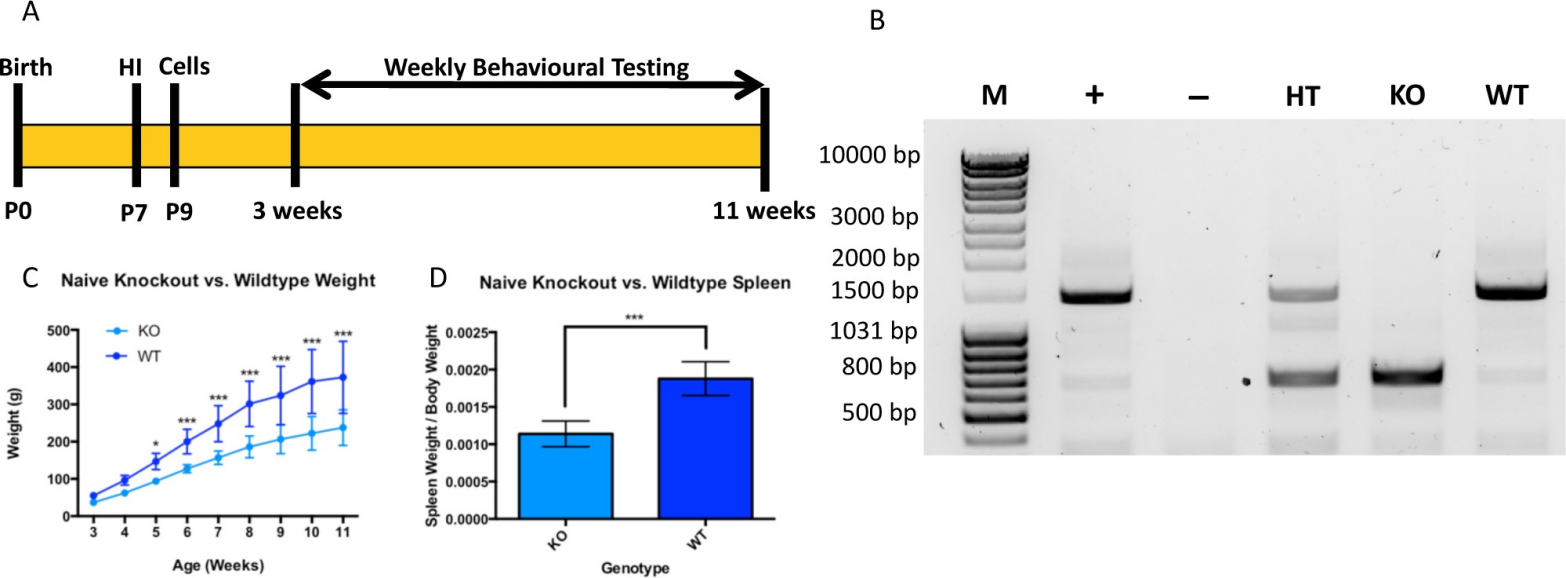
Study design and *Prkdc* KO characteristics. (A) Study design. Two cohorts of animals were used, one for characterization of the injury model, and one that was used to study cell transplant survival. Time scale is according to animal age. (B) Genotyping for the *Prkdc* gene. From left to right, DNA ladder (M), positive control Long Evans rat (+), negative control ddH_2_O (-), HT rat (1627 kDa and 872 kDa), KO rat (872 kDa), and WT rat (1627 kDa). (C) Growth trajectories are unique across *Prkdc* KO and WT rats. (D) Spleen size is significantly smaller in uninjured *Prkdc* KO rats. Error bars = ±SD. * p<0.05, ***p<0.001.

All animals used in the study were maintained under pathogen-free conditions with a 12-hr day/night light cycle and provided with access to rodent chow and water *ad libitum*. Breeding pairs were established between *Prkdc* heterozygous (HT) dams and *Prkdc* HT or KO males (original breeders obtained from Horizon Labs). Dams of the HT genotype were used due to high mortality rates in KO mothers prior to, or following the birth of pups. No more than one female and one male rat were housed per cage. Several days prior to parturition, dams were separated from their mates and provided with nesting materials in order to prepare for delivery. Date of birth was recorded and considered P0, and pups were weaned at P21. Both male and female animals were used in the study. No experimental animals were housed alone in order to prevent social isolation from having an effect on wellness, as well as on performance during behavioural testing. Prior to weaning, animals were monitored daily to see that they were mobile (age-appropriate) and feeding from the dam. After weaning, animals were monitored daily for signs of illness (e.g. ruffled fur, inactivity, significant lack of weight gain which was measured weekly). If animals were identified to have an untreatable illness before the P77 endpoint, they were euthanized to prevent further suffering. Only 2 animals became sick after weaning (and close to endpoint), likely due to the SCID phenotype. All procedures were approved by the Animal Care Committee at the University Health Network (Animal Use Protocol 2126) in accordance with the Canadian Council on Animal Care. A schematic of the study design is presented in Fig 1A.

### Genotypic and phenotypic characterization of rats

Polymerase chain reaction (PCR) was used to genotype animals according to the presence of the mutated (KO; 872 kDa) and intact (WT; 1627 kDa) *Prkdc* gene. Animals from KO and WT genotypes were randomly divided into Naïve, Sham, HI, and HI+NPC groups. All animals were given blinding IDs so as to minimize experimental bias. However, as animals aged, weight differences between KO and WT animals became obvious, which prevented the experimenter from being blinded to the genotype during behavioural testing. Animals were weighed at weekly time-points to confirm weight differences between Naïve KO and WT animals. At study endpoint, the spleens of animals were removed, weighed, and compared between Naive KO and WT animals to confirm the genotyping results with findings from Mashimo et al. [25]. Sample size for weight assessment was n = 12 (KO) and n = 10 (WT). Sample size for spleen size assessment was n = 11 (KO) and n = 8 (WT).

### HI brain injury model

At P7, the right common carotid artery was completely cauterized under 3% isoflurane anesthetic and on a heating pad. Prior to suturing, 2–3 drops of bupivacaine (analgesic) were administered at the incision site. All surgeries were performed in less than 10 minutes. Following recovery from surgery, pups were returned to the dam to recover for 45 minutes. Combined surgery time for all animals did not exceed 1 hour in order to reduce variability in injury caused by variations in post-operative recovery times. Pups were then placed inside glass jars (400mL, maximum 2 animals/jar) that were partially submerged in a 36.5±0.5°C water bath for 15 minutes in order to acclimatize body temperature to prevent any neuroprotective effects of hypothermia. Therefore, total post-operative recovery time was 1 hour. Pups then underwent 75 minutes of systemic hypoxia (8% O_2_/92% N_2_) at 36.5±0.5°C, followed by 15 minutes in the jars exposed to normoxic air. Pups were then returned to their dams until P21 at which time they were weaned. Sham animals underwent all surgical procedures without cauterization of the artery. Naïve animals served as genotype controls and did not undergo any procedures.

### Generation of human-derived neural precursor cells

The BC1 hiPSC line was provided by the Centre for Commercialization of Regenerative Medicine (CCRM, Toronto, Canada). hiPSCs were maintained in mTeSR1 culture media (STEMCELL Technologies, Vancouver, Canada) and acclimated to the same culture conditions for several passages. hiPSCs were differentiated to NPCs using dual SMAD inhibition in monolayer culture with some modifications. Partially differentiated colonies were manually removed before differentiation. At the start of differentiation (day 0), hiPSCs were dissociated to single cells and re-plated as a monolayer on Matrigel (Corning, Tewksbury, MA) at a density of 20,000 cells/cm^2^ in mTeSR1 media. After cells reached 90% confluency, media was gradually changed over two days to neural induction media (NIM) consisting of a 1:1 ratio of DMEM:F12 media supplemented with B27, N2, FGF (10ng/ml), 10μM TGFβ inhibitor (STEMCELL Technologies, SB431542), and Noggin (200ng/ml). After 7 days in culture, neural rosettes were manually isolated and plated as single cells on poly-L-lysine (PLL)/Laminin coated dishes in neural expansion media (NEM) consisting of neurobasal media supplemented with B27, N2, FGF (10ng/ml) and EGF (20ng/ml) for two passages. The resulting cells were then cultured in NEM as single cells on Ultra-Low adherent dishes (Corning, Tewksbury, MA) at a density of 10,000 cells/ml to form primary neurospheres. After 5 days in culture, each individual clonal neurosphere was separately plated into a well of a PLL/Laminin coated 24-well plate in order to proliferate. These steps were then repeated to obtain secondary clonal neurospheres. For expansion of the culture, secondary clonal neurospheres were cultured in NEM on PLL/Laminin. During the period of induction, which took over 2 weeks, the cells progressed through the neural rosette and neurosphere stages.

hiPSC-NPCs were stably transfected with a *piggyBac* vector to express enhanced green fluorescent protein (eGFP). The *piggyBac* vector carried the eGFP and puromycin-N-acetyl-transferase (pac) genes and was stably transfected into hiPSC-NPCs using the Amaxa nucleofection method. After transfection, cells were allowed to recover for 5 days, and selection was started with medium containing 1 μg/mL Puromycin (Sigma Aldrich, Saint Louis, MO). Single clones were selected and cultured until clonal lines were established.

The Neurosphere Assay was used to demonstrate the ability of hiPSC-NPCs to generate neurospheres. Neurospheres and monolayer cultures of hiPSC-NPCs were immunocytochemically stained with the neural progenitor markers Nestin (Rabbit polyclonal, Millipore ABD69; dilution 1:500) and Pax6 (Mouse monoclonal, Millipore MAB5553; dilution 1:500) to confirm their identity. Cells were fixed for 20 min with 4% paraformaldehyde (PFA) in 1x phosphate-buffered saline (PBS) and 40% sucrose at room temperature. Following fixation, cells were permeabilized with 0.1% Triton X-100 and 0.1% sodium citrate in PBS for 5 min and then placed in blocking buffer containing 5% bovine serum albumin (BSA) for 1 hour. Primary antibodies were diluted in the blocking buffer solution and incubated with the cells overnight at 4°C. The following day, cells were washed extensively in 1x PBS, and then incubated with DAPI and fluorophore-conjugated secondary antibodies (Alexa Fluor 594 goat-anti rabbit A11012 and Alexa Fluor 594 goat anti-mouse R37121, ThermoFisher; dilution 1:1000) for 1 hour at room temperature. The secondary antibodies were again washed extensively in 1x PBS and coverslipped. Cells were imaged using a Nikon *Eclipse Ti* Lumencor epifluorescent microscope.

In order to study the differentiation potential of hiPSC-NPCs *in vitro*, hiPSC-NPCs were dissociated into single cells and plated on poly-ornithine/laminin coated cover glasses (24 well plates: 4 ×10^3^ cells/well). Cells were grown in Neurobasal medium (Thermofisher Scientific, 21103049) supplemented with N2 (Thermofisher Scientific, 17502048), B27 (Thermofisher scientific, 17504044), 0.1% fetal bovine serum (FBS), 10 μM Forskolin (STEMCELL Technologies, 72112) and glutamax (Thermofisher scientific, 21103049) for 10 days. Immunocytochemical staining was performed as described above, for neurons (βIII-Tubulin, mouse monoclonal, Millipore MAB1637; dilution 1:1000), astrocytes (GFAP, mouse monoclonal, Millipore MAB360; dilution 1:1000) and oligodendrocytes (O1, mouse monoclonal, Millipore MAB344; dilution 1:500).

### Cell transplantation

After 6 passages, hiPSC-NPCs were harvested and prepared at a density of 50,000 cells/μl and placed on ice. HI-injured pups were anesthetized at P9 under 3% isoflurane and a midline incision was made into the skin dorsal to the skull. A stereotactic frame (Kopf Instruments) was used to landmark the precise region of injection (1mm caudal, 3mm lateral, 3mm ventral; relative to Bregma). A hole was drilled into the skull. Isoflurane was then changed to 1.5–2% and a fine metal needle was lowered into the caudate-putamen (CPu). The disturbed tissue was allowed to settle around the needle for 1 minute, and then 2μl of hiPSC-NPCs (~100,000 cells) was injected at a rate of 400 nl/min (~5 min for injection). After injection, the needle was left in place for 2.5 minutes before slowly being retracted. Isoflurane was raised to 3%, and sterile saline was gently sprayed onto the surface of the skull to allow the skin to be pulled toward the midline with ease. 5–0 silk sutures were then used to close the skin. Animals were administered saline according to body weight (<0.02mL/g). Once pups recovered under a heat lamp, they were injected with 0.1mL of buprenorphine to minimize the experience of post-operative pain. Pups were then returned to the dam, weaned at P21, and allowed to survive until 10 weeks post-transplant.

### Cell survival assessment

Rats that received hiPSC-NPCs did not undergo behavioural testing as their purpose was purely to assess human cell survival. At 10 weeks post-transplant animals were transcardially perfused with PFA and the tissue was processed as described below. 20μm sections were placed onto glass slides, and sections containing the injection site epicentre (i.e. the bulk of eGFP-positive transplanted cells) were identified. The sections were counterstained with DAPI and coverslipped, as described below. Z-stacked images of the transplanted cells were taken using a Nikon C2 Confocal *Eclipse Ti* microscope and used to create a maximum intensity projection image. eGFP-positive cells that co-localized with DAPI were counted manually using ImageJ across 3 serial sections. The average number of eGFP-positive cells per section at the injection site epicentre is reported. Sample size was n = 6 (KO) and n = 4 (WT).

### Behavioural testing

Testing of sensorimotor deficits began at P21 (2 weeks after injury) using the cylinder-rearing test. Tests were performed weekly until 10 weeks after injury, at which time the animals were sacrificed. The cylinder-rearing test consisted of animals exploring a Plexiglass cylinder independently for 5 minutes. Trials were not included in the analysis if an animal did not touch the cylinder walls at least 10 times (only a concern between P21-P28). Animals were assessed on their relative use of the left (injured) and right (uninjured) forelimbs. The pasta-handling test was used as a measure of manual dexterity, relying on both sensory and motor function. Animals were given three trials to eat standardized pieces of vermicelli pasta (6.5cm; Italpasta, Brampton, ON, Canada) and the time taken to eat the pasta was averaged for each animal. Testing was performed in the home cage in order to minimize anxiolytic behaviours. Depending on the analysis, sample size varied slightly. The sample size for Naïve animals was n = 9–12, for Sham animals was n = 11–14, and for HI animals was n = 13–18. Detailed sample size information can be found in S1 Table.

### Tissue processing

Animals were sacrificed at 10 weeks after injury. Briefly, animals were deeply anesthetized with 5% isoflurane and transcardially perfused with 180mL of cold 1x PBS. After removing the spleen for weighing, animals were perfused with 120mL of cold 4% PFA, pH 7.4. The brain was removed and post-fixed in 10% sucrose in PFA for 4 hours at 4°C. Following post-fixation, brains were washed twice in cold 1x PBS and then cryoprotected in 20% sucrose in 1x PBS overnight at 4°C. Brains were then embedded in Optimal Cutting Temperature (OCT) media and stored at -80°C until cryosectioning. Brains were sectioned at 20μm per section from the level of the genu of the corpus callosum until the splenium of the corpus callosum (inclusive).

### Histopathology

Slides were processed for histopathological analysis by staining with luxol fast blue (LFB) and hematoxylin & eosin (HE). Stereology and Cavalieri estimation were performed at 10x magnification on a Leica DMRB microscope using Stereo Investigator software (MBF Bioscience) to assess the area of the lateral ventricles and hippocampus. 5x magnification was used to assess the area of the internal capsule, and the thickness of the corpus callosum and cortex. Locations used for thickness measurements in each region of interest (ROI) were held constant across all animals by relying on anatomical landmarks. On average, 6–10 sections were analyzed per animal. All regions were compared as a ratio of the right (injured) hemisphere structure, divided by the left hemisphere structure (uninjured), multiplied by 100% (*R*/L*100%). Note that four KO HI and two WT HI animals were excluded from the analysis due to complete liquefaction of the right hemisphere. Depending on the analysis, sample size varied slightly. The sample size for Naïve animals was n = 6–7, for Sham animals was n = 6–7, and for HI animals was n = 10–11. Detailed sample size information can be found in S1 Table. Representative stitched brain images were taken on the same Leica DMRB microscope at 5x magnification.

### Immunohistochemistry

Slides were processed for immunohistochemical analysis by staining for neurons, astrocytes, microglia, and oligodendrocytes. Briefly, slides were washed with 1x PBS (1x10min) and then blocked in 5% skim milk containing 1% BSA and 0.3% Triton X-100 (NeuN/GFAP), or in 5% goat serum containing 0.3% Triton X-100 (Iba-1), or in 10% goat serum containing 0.3% Triton X-100 (Olig2) for 1 hour. Slides were then incubated with antibodies against neurons (NeuN, mouse monoclonal, Millipore MAB377; dilution 1:500), astrocytes (GFAP, rabbit polyclonal, Millipore AB5804; dilution 1:1000), microglia (Iba-1, rabbit monoclonal, Abcam, ab178847; dilution 1:500), and oligodendrocytes (Olig2, rabbit polyclonal, Millipore AB9610; dilution 1:1000) at 4°C overnight. The following day, slides were washed with 1x PBS (3x10min) and incubated with secondary antibodies (DAPI D3571, Alexa Fluor 488 goat anti-mouse A11001, Alexa Fluor 488 goat anti-rabbit A11034, Alexa Fluor 568 goat anti-rabbit A11011, Invitrogen; dilution 1:500) for 1 hour at room temperature out of the light. Slides were then washed again (3x10min) and coverslipped with Mowiol Mounting Media. Images for neuronal counts in the primary motor cortex (M1), primary somatosensory cortex (S1) and hippocampus (CA1 and CA3 pyramidal layers) were taken at 20x magnification on a Zeiss Axioplan2 epifluorescent microscope using AxioVision 4.6 software. Locations used for counting in each ROI were held constant across all animals by relying on anatomical landmarks. Automated counting of each ROI was validated and performed using ImageJ for all cortical regions, and manual counting was performed for hippocampal regions by a blinded experimenter. Manual counts for oligodendrocytes (Olig2+) were performed by a blinded experimenter on landmarked images of the corpus callosum taken at 20x magnification on the same microscope. Quantitative gliosis assessment was performed by calculating the percent area of GFAP-positive stain and Iba-1-positive stain in each image taken at 20x magnification on a Nikon C2 Confocal *Eclipse Ti*. Z-stacks were taken for each region assessed (S1, CPu, and thalamus) and combined as a maximum intensity projection prior to analysis. Similar to histopathological analyses, all regions were compared as a ratio of the right hemisphere structure, divided by the left hemisphere structure, multiplied by 100% (*R*/L*100%). A minimum of 3 sections were evaluated for each animal for all assessments. Four KO HI and two WT HI animals were excluded from the analysis due to complete liquefaction of the right hemisphere. Depending on the analysis, sample size varied slightly. The sample size for Naïve animals was n = 4–7, for Sham animals was n = 5–7, and for HI animals was n = 6–9. Detailed sample size information can be found in S1 Table. All representative immunohistochemical images were taken at 20x and 60x magnification on a Nikon C2 Confocal *Eclispe Ti*.

### Statistical analysis

Data were analyzed using GraphPad Prism Software (Version 6). Histological analyses for within-genotype comparisons were done by using a one-way ANOVA followed by Bonferroni’s post-hoc test, comparing Naïve, Sham, and HI groups. Between-genotype comparisons were done by normalizing Sham and HI animals to their respective genotypically-similar Naïve counterparts, followed by a one-way ANOVA with pairwise comparisons (Sham KO vs. Sham WT and HI KO vs. HI WT), followed by Bonferroni’s post hoc test. Where data did not follow a Gaussian distribution, a nonparametric Kruskal-Wallis test followed by Dunn’s post-hoc test was performed. All within-genotype behavioural testing was analyzed using a two-way ANOVA followed by Bonferroni’s post-hoc test. Between-genotype comparisons were done by normalizing Sham and HI animals to their respective genotypically-similar Naïve counterparts, followed by a two-way ANOVA with pairwise comparisons (Sham KO vs. Sham WT and HI KO vs. HI WT), followed by Bonferroni’s post hoc test. Weight data (Fig 1C) was also analyzed using a two-way ANOVA, followed by Bonferroni’s post-hoc test. Assessment of differences in spleen weight of KO and WT animals, as well as cell survival assessments, was done using a Mann-Whitney t-test. Statistical significance was defined as p<0.05. Error bars are reported as mean ± standard deviation (SD) unless stated otherwise.

## Results

### *Prkdc* knockout rats are phenotypically different from wildtype rats

Genotyping for the *Prkdc* gene using PCR consistently revealed a 1627 kDa band for WT animals, a 1627 kDa and mutated 872 kDa band for heterozygotes (HT), while only the mutated 872 kDa band was visible in KO animals (Fig 1B). Further differences between Naive KO and WT animals were observed in weight up to 11 weeks of age (Fig 1C) and after euthanasia, the unfixed spleens of Naive KO animals were observed to be significantly smaller than those of WT animals after accounting for body weight (Fig 1D). HI brain injury led to a 24.1% mortality rate in KO pups, while WT pups had an 11.8% mortality rate.

### HI-Injured *Prkdc* knockout rats display sensorimotor deficits that persist over time

**Fig 2:**
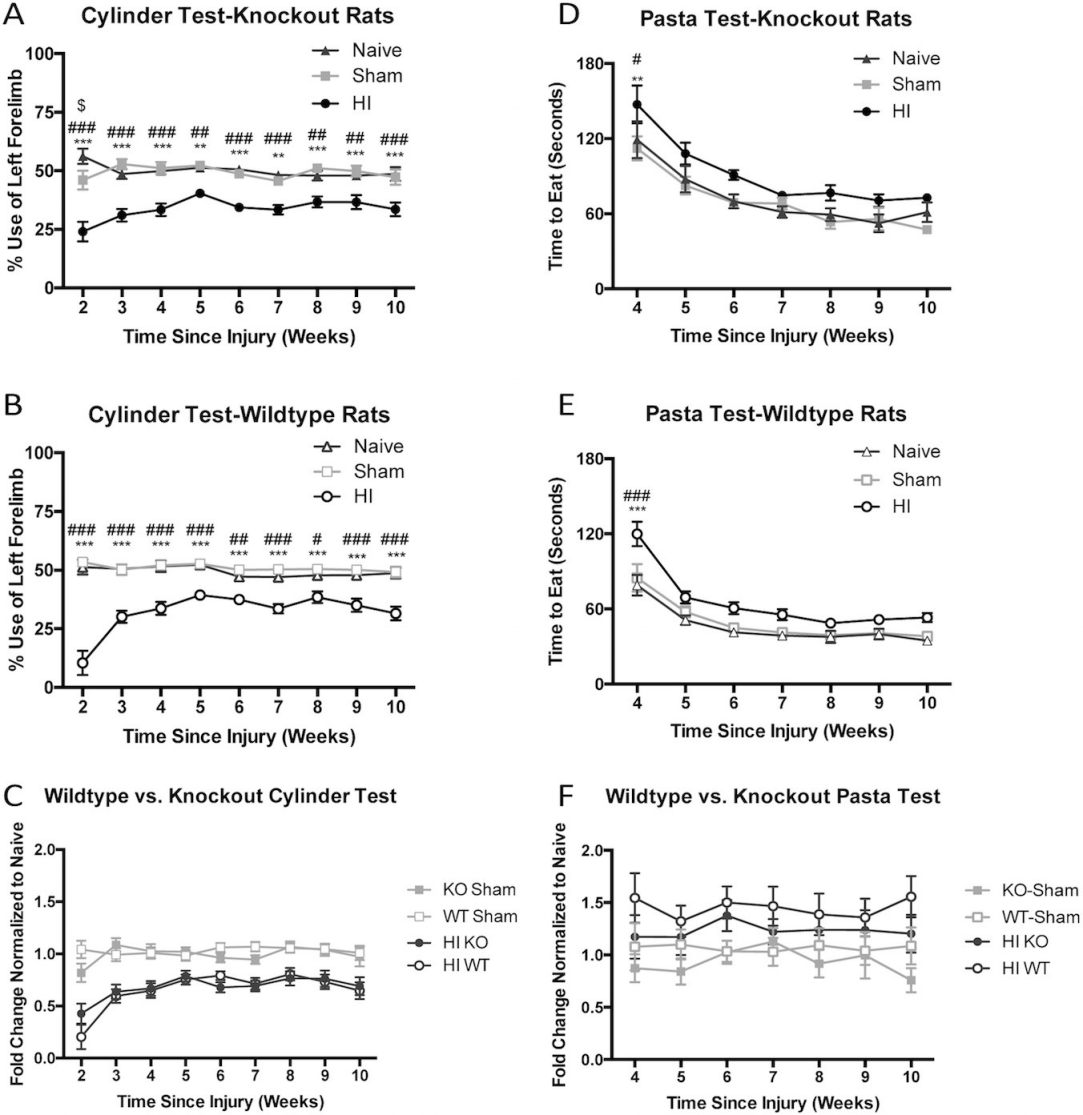
Behavioural outcomes. Both *Prkdc* KO (A) and WT (B) injured animals are impaired in the use of their left forelimb when compared to Naïve and surgical Sham animals. There is no difference in performance of injured animals on the cylinder test when compared across genotypes (C). Both KO (D) and WT (E) HI animals take significantly longer to complete the pasta test at 4 weeks after injury, but perform similarly to uninjured animals after this time-point. There is no difference in performance of injured animals on the pasta test when compared across genotypes (F). Error bars = ±SEM. # p<0.05, ## p<0.01, ### p<0.001 Naïve vs. HI; ** p<0.01, ***p<0.001 Sham vs. HI.

The cylinder test was used as a measure of sensorimotor function. The test is sensitive, reliable, and easy to administer, especially in rats as young as P21. HI brain injury in the right hemisphere led to significantly reduced use of the left forelimb during exploration of the cylinder in both KO and WT genotypes when compared to controls. These differences were obvious at 2 weeks following injury and persisted until endpoint at 10 weeks following injury (Fig 2A and 2B). There was no effect of genotype on cylinder test performance (Fig 2C). The pasta test revealed mild sensorimotor deficits in both KO and WT animals at 4 weeks following injury, but clear differences between injured and control animals disappeared after this time-point (Fig 2D and 2E). There was no effect of genotype on performance on this test (Fig 2F).

### Grey and white matter structures are affected by HI-injured *Prkdc* knockout rats

Cavalieri estimation, cell counts, and gliosis quantification in various brain structures at 10 weeks following injury revealed reductions in area, thickness, and cell numbers, as well as increased gliosis in injured animals when compared to control animals.

#### Primary somatosensory and motor cortices

**Fig 3:**
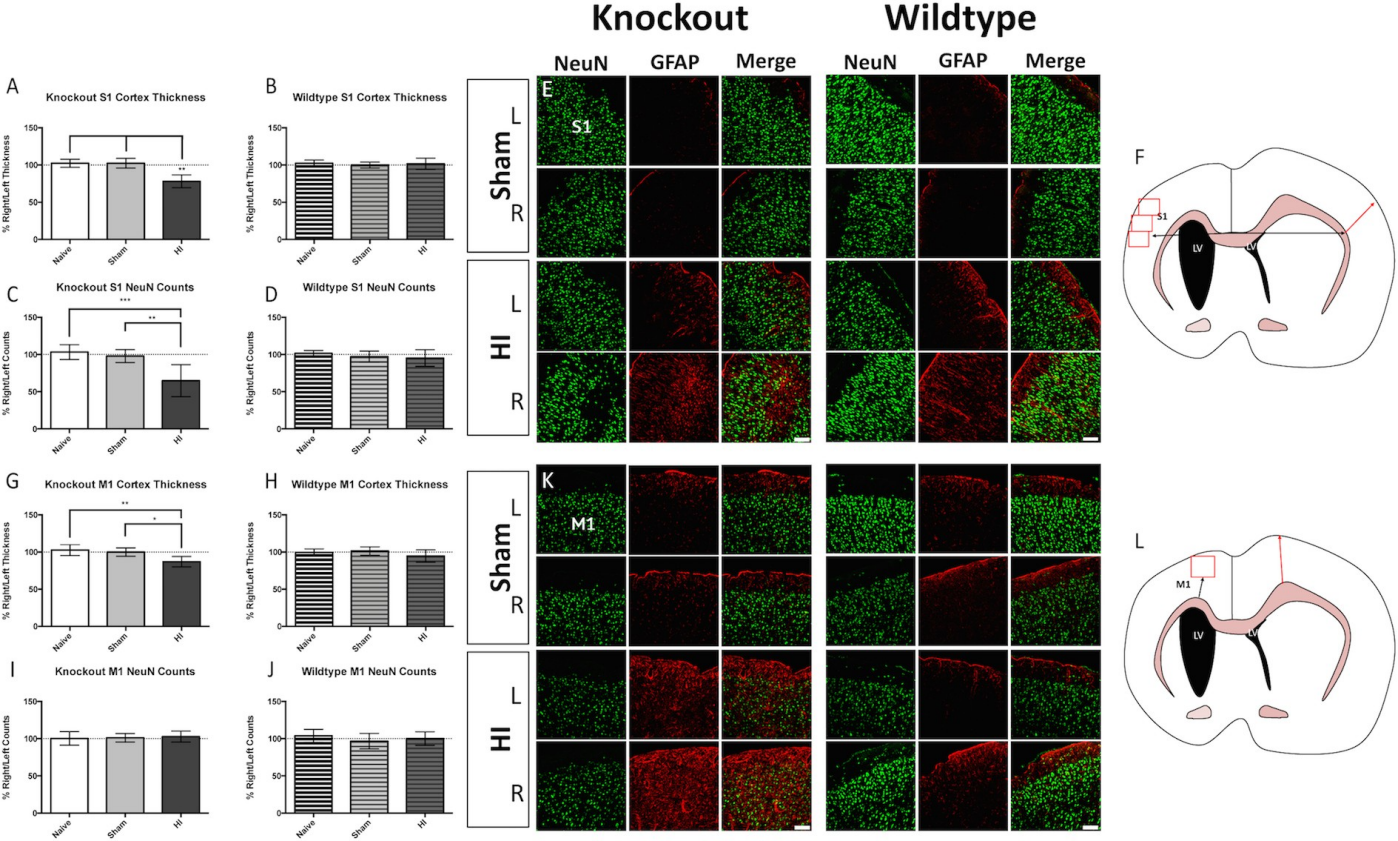
Cortical injury. Injured *Prkdc* KO rats display reductions in the thickness of the right S1 (A) while their WT counterparts do not (B). Injured KO rats also display a concomitant reduction in neurons in the right S1 (C) while WT animals do not (D). Representative immunofluorescence in S1 of KO Sham (E top left), KO injured (E bottom left), WT Sham (E top right), and WT injured (E bottom right) in the right and left hemispheres. Where neuronal loss was observed, astrogliosis was evident (green = NeuN, red = GFAP). (F) Landmarking for the S1 measurements was performed from the midline (black arrows) and locations for the S1 thickness measurement (red arrow) and S1 neuronal counts (red boxes) are delineated. Injured *Prkdc* KO rats display reductions in the thickness of the right M1 (G) while their WT counterparts do not (H). Neither KO (I) nor WT (J) injured animals display neuronal loss in M1. Representative immunofluorescence in M1 of KO Sham (K top left), KO injured (K bottom left), WT Sham (K top right), and WT injured (K bottom right) in the right and left hemispheres (green = NeuN, red = GFAP). (L) Landmarking for the M1 measurements was performed from the peak of the cingulum, and locations for the M1 thickness measurement (red arrow) and M1 neuronal counts (red box) are delineated. Scale bars = 100μm, 20x. Error bars = ±SD. * p<0.05, ** p<0.01, ***p<0.001.

A reduction in right cortical thickness was apparent at the site of S1 in KO injured animals (Fig 3A), but not in WT injured animals (Fig 3B). In line with these results, KO injured animals exhibited neuronal loss in the right S1 (Fig 3C and 3E left panel), while WT animals did not exhibit this aspect of the injury (Fig 3D and 3E right panel). Measurement locations are delineated in Fig 3F. A reduction in the right cortical thickness was apparent at the site of M1 in KO injured animals (Fig 3G), but not in WT injured animals (Fig 3H). There were no changes in neuronal density in M1 of both injured KO (Fig 3I and 3K left panel) and WT animals (Fig 3J and 3K right panel). Measurement locations are delineated in Fig 3L.

#### Hippocampus

**Fig 4:**
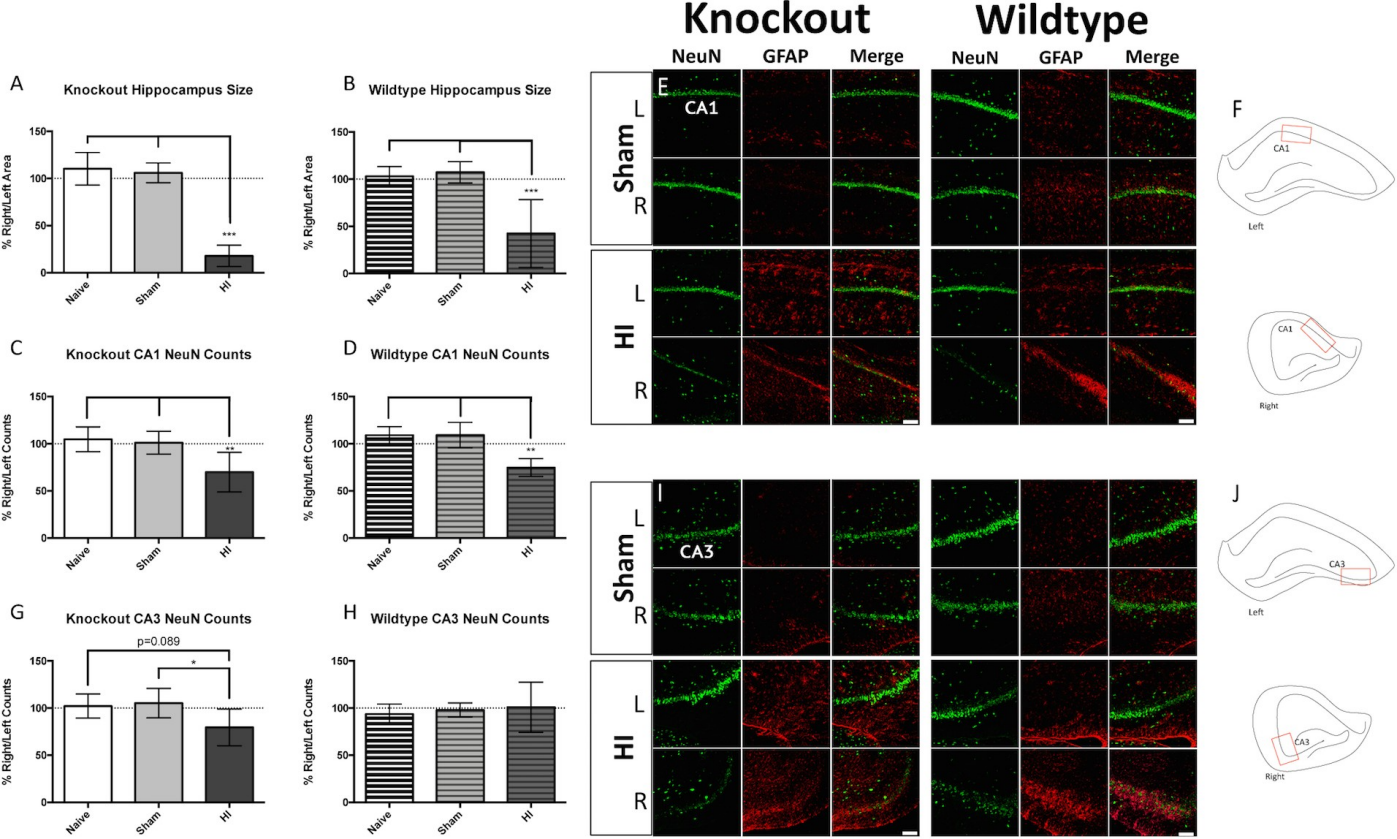
Hippocampal injury. Injured *Prkdc* KO (A) and WT (B) rats both exhibited reductions in right hippocampal area. Both KO (C) and WT (D) injured rats also display reductions in the number of neurons in the right CA1 pyramidal layer. Representative immunofluorescence in CA1 of KO Sham (E top left), KO injured (E bottom left), WT Sham (E top right), and WT injured (E bottom right) in the right and left hemispheres. Where neuronal loss was observed, astrogliosis was evident (green = NeuN, red = GFAP). (F) Measurement locations for CA1 neuronal counts (red boxes) are shown for the left and right hemispheres; A trend toward reduction in neurons in the right CA3 pyramidal layer was observed in KO (G), but not in WT (H) injured rats. Representative immunofluorescence in CA3 of KO Sham (I top left), KO injured (I bottom left), WT Sham (I top right), and WT injured (I bottom right) in the right and left hemispheres. Gliosis is also evident in these injured animals (green = NeuN, red = GFAP). (J) Measurement locations for CA3 neuronal counts (red boxes) are shown for the left and right hemispheres; Scale bars = 100μm, 20x. Error bars = ±SD. * p<0.05, ** p<0.01, ***p<0.001.

The hippocampus is an important structure for learning and memory, and it is impacted by neonatal HI brain injury [29–31]. Both KO and WT animals displayed significant reductions in right hippocampal area when compared to uninjured controls (Fig 4A and 4B). Both groups also displayed significant reductions in neurons in CA1 (Fis 4C, 4D and 4E). Measurement locations for the CA1 pyramidal counts are depicted in Fig 4F for the right and left hemispheres. Only KO injured animals displayed a trend toward neuronal loss in the CA3 pyramidal layer (Fig 4G and 4I left panel), while WT injured animals did not (Fig 4H and 4I right panel). Measurement locations for the CA3 pyramidal counts are depicted in Fig 4J for the right and left hemispheres.

#### Gliosis

**Fig 5:**
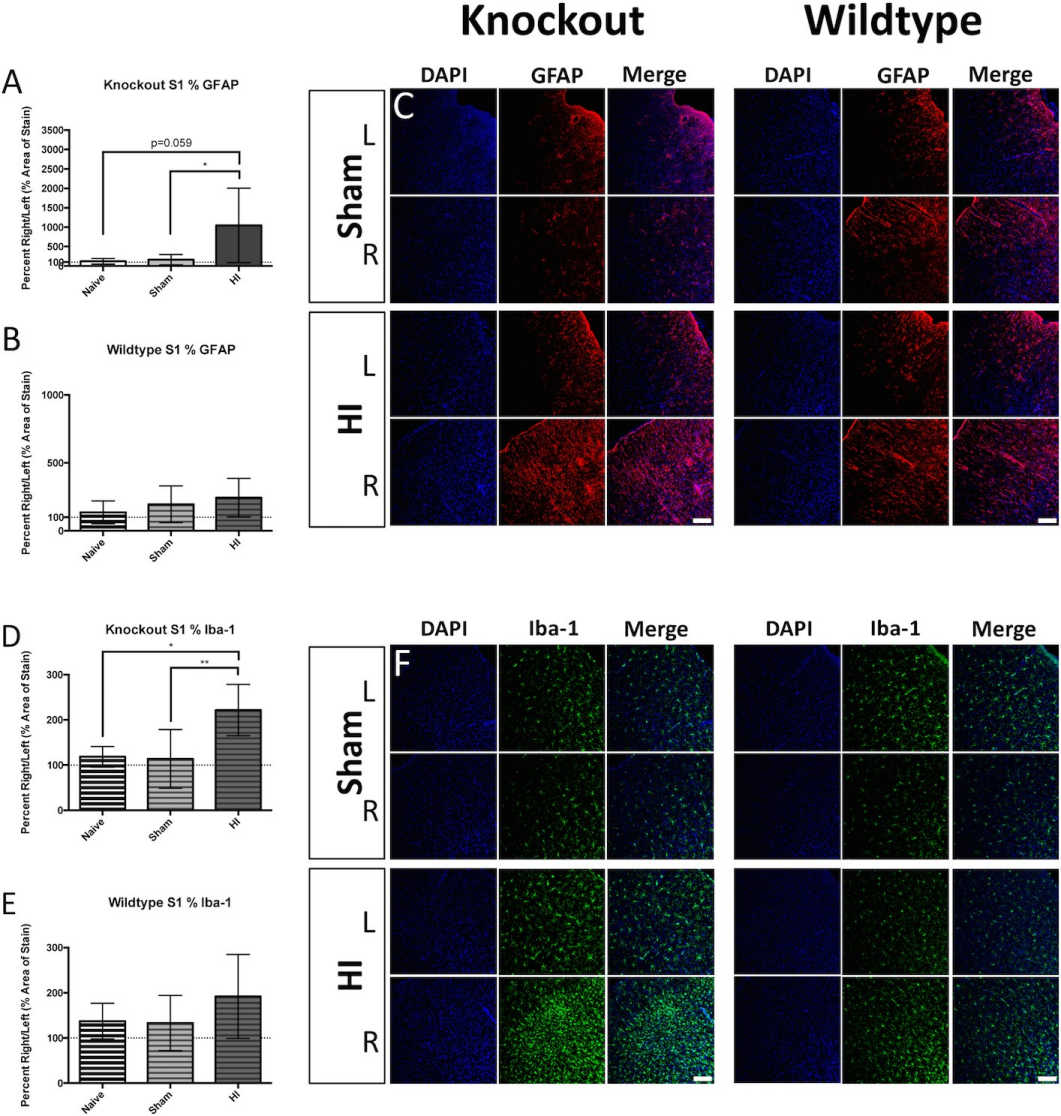
Somatosensory cortex gliosis. KO injured animals displayed a trend toward elevated stain area occupied by GFAP-positive signal when compared to uninjured controls (A), while WT injured animals did not (B). Representative immunofluorescence is depicted for KO Sham (C top left), KO injured (C bottom left), WT Sham (C top right), and WT injured (C bottom right) in the right and left hemispheres (blue = DAPI, red = GFAP). Percent area occupied by Iba-1-positive staining was elevated in KO injured animals when compared to uninjured controls (D), while stain area was not elevated in WT injured animals (E). Representative immunofluorescence is depicted for KO Sham (F top left), KO injured (F bottom left), WT Sham (F top right), and WT injured (F bottom right) in the right and left hemispheres (blue = DAPI, green = Iba-1). Scale bars = 100μm, 20x. Error bars = ±SD. * p<0.05, ** p<0.01.

**Fig 6:**
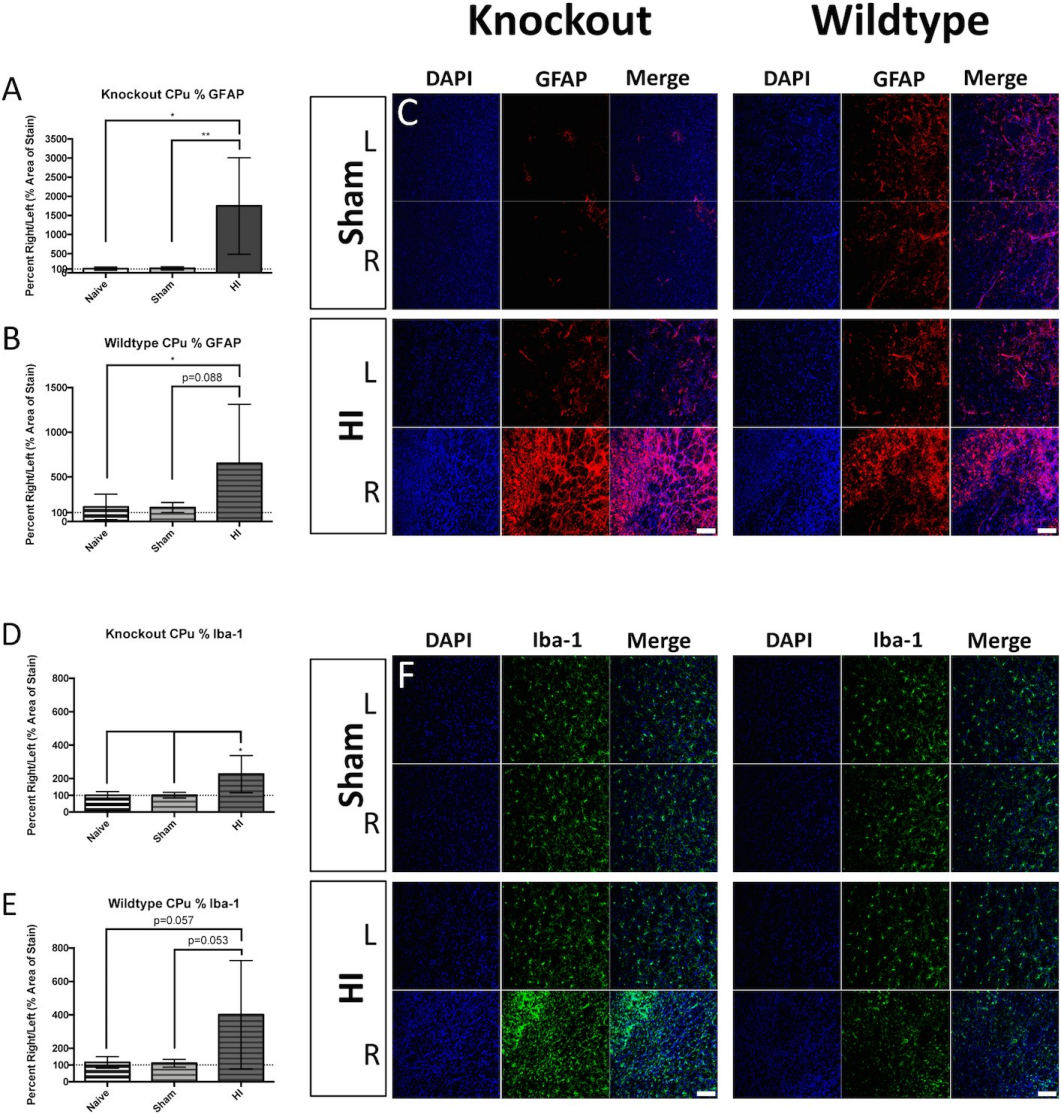
Caudate-putamen gliosis. KO injured animals displayed a significant increase in the percent area occupied by GFAP-positive signal when compared to uninjured controls (A), and WT injured animals trended toward similar results (B). Representative immunofluorescence is depicted for KO Sham (C top left), KO injured (C bottom left), WT Sham (C top right), and WT injured (C bottom right) in the right and left hemispheres (blue = DAPI, red = GFAP); Percent area occupied by Iba-1-positive staining was elevated in KO injured animals when compared to uninjured controls (D), and WT injured animals trended toward similar results (E). Representative immunofluorescence is depicted for KO Sham (F top left), KO injured (F bottom left), WT Sham (F top right), and WT injured (F bottom right) in the right and left hemispheres (blue = DAPI, green = Iba-1); Scale bars = 100μm, 20x. Error bars = ±SD. * p<0.05, ** p<0.01.

**Fig 7:**
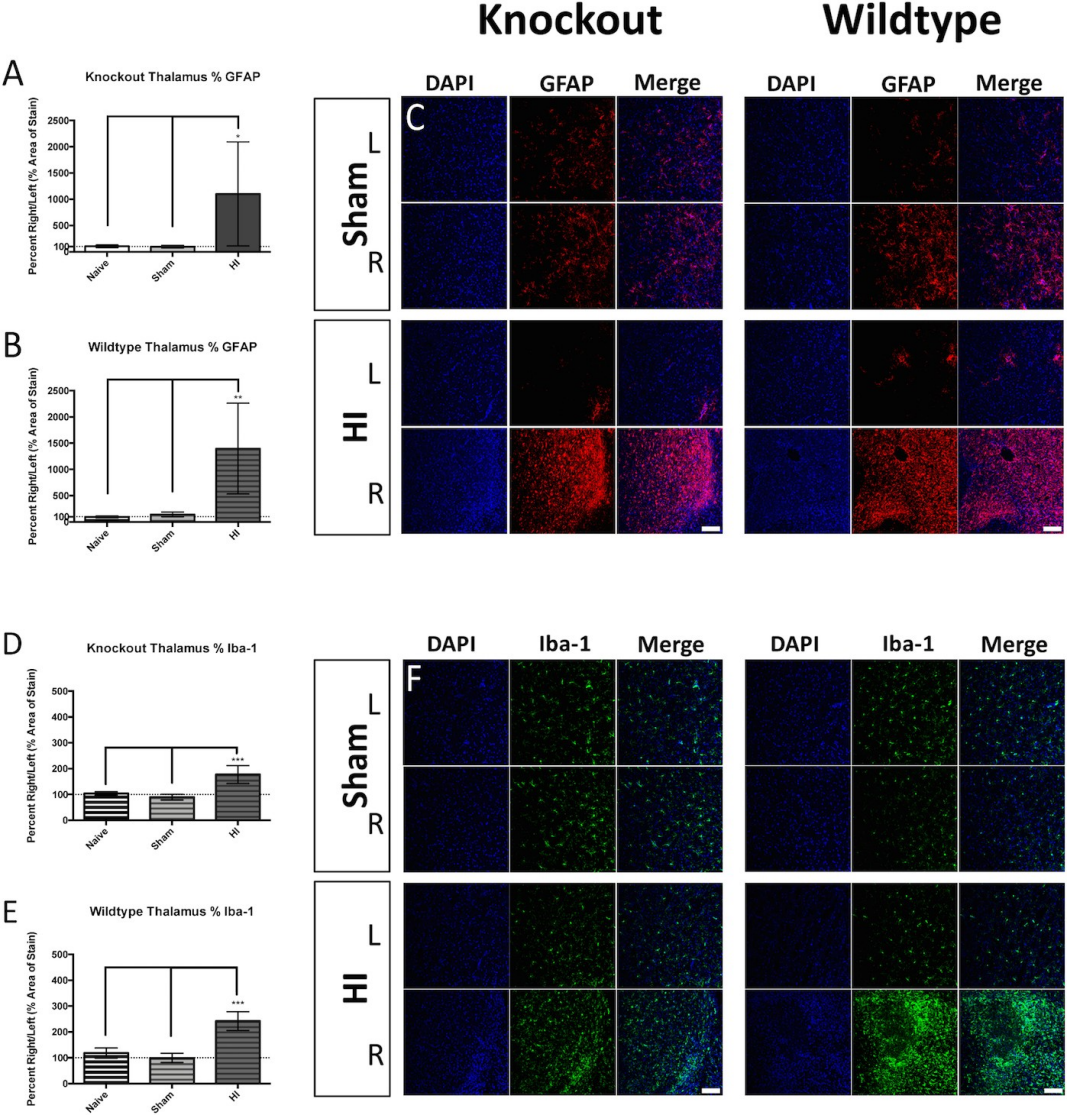
Thalamus gliosis. KO injured (A) and WT injured (B) animals displayed a significant increase in the percent area occupied by GFAP-positive signal when compared to uninjured controls. Representative immunofluorescence is depicted for KO Sham (C top left), KO injured (C bottom left), WT Sham (C top right), and WT injured (C bottom right) in the right and left hemispheres (blue = DAPI, red = GFAP). Percent area occupied by Iba-1-positive staining was elevated in both KO injured (D) and WT injured (E) injured animals when compared to uninjured controls. Representative immunofluorescence is depicted for KO Sham (F top left), KO injured (F bottom left), WT Sham (F top right), and WT injured (F bottom right) in the right and left hemispheres (blue = DAPI, green = Iba-1). Scale bars = 100μm, 20x. Error bars = ±SD. * p<0.05, ** p<0.01, *** p<0.001.

Examination of the brain revealed gliosis in various grey matter regions. The percent area of GFAP-positive signal trended toward increased levels in the S1 of KO injured animals (Fig 5A and 5C left), but not in WT injured animals (Fig 5B and 5C right panel). The percent area occupied by Iba-1-positive signal was significantly increased in the S1 of KO injured animals (Fig 5D and 5F left panel), but not in WT injured animals (Fig 5E and 5F right panel). The percent area of GFAP-positive signal was significantly increased in the CPu of KO injured animals (Fig 6A and 6C left panel), and was trending in WT injured animals (Fig 6B and 6C right panel). The percent area occupied by Iba-1-positive signal in the CPu was significantly increased in KO injured animals (Fig 6D and Fig 6F left panel), and was trending in WT injured animals (Fig 6E and Fig 6F right panel). Examination of the thalamus also revealed elevated percent area occupied by GFAP-positive staining in both KO injured (Fig 7A and Fig 7C left panel), and WT injured animals (Fig 7B and Fig 7C right panel). The percent area occupied by Iba-1-positive signal in the thalamus was also significantly elevated in KO injured animals (Fig 7D and Fig 7F left panel), and in WT injured animals (Fig 7E and Fig 7F right panel).

#### Corpus callosum

**Fig 8:**
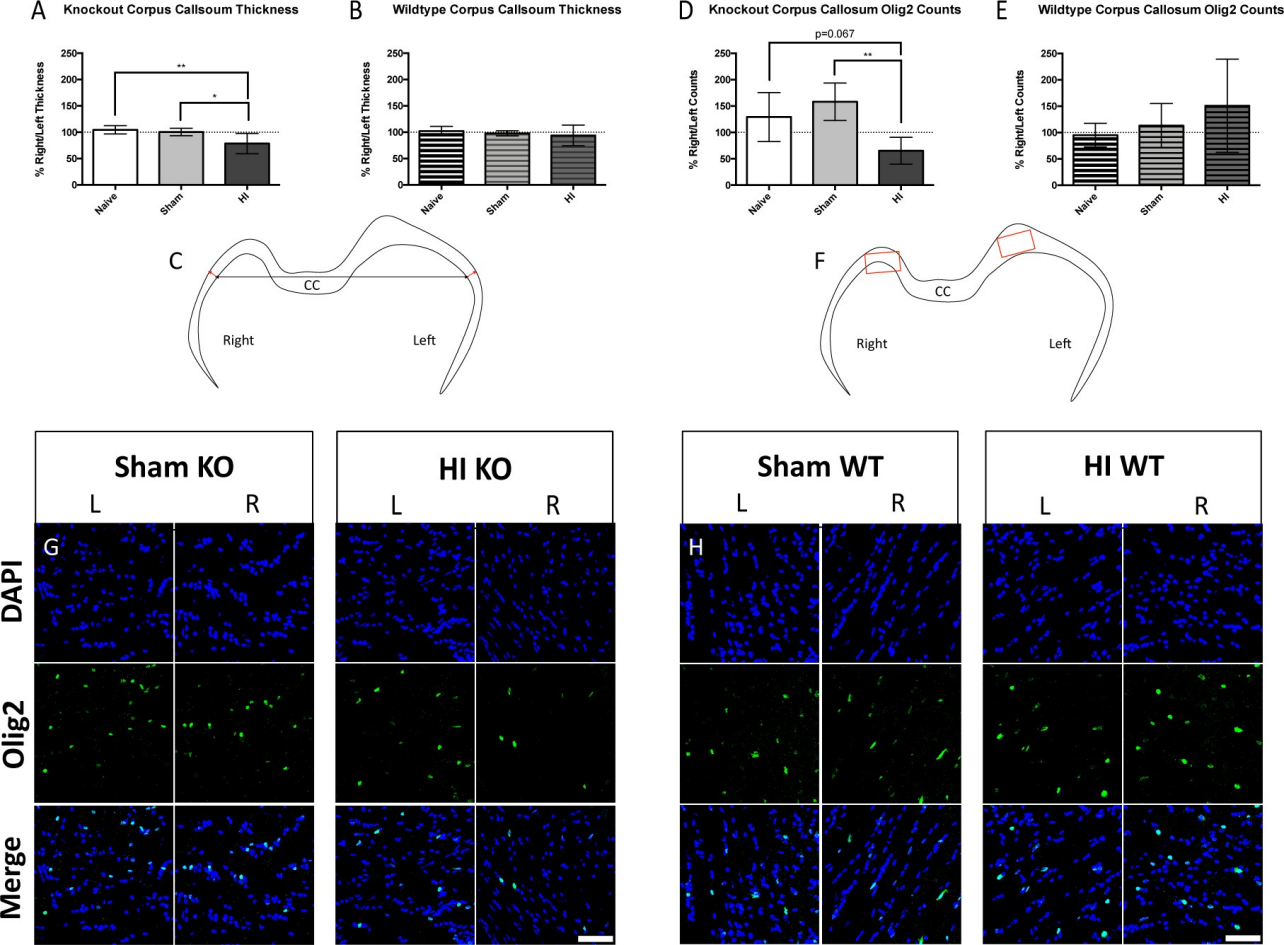
Corpus callosum injury. KO (A) but not WT (B) injured animals displayed reductions in thickness of the corpus callosum in the right hemisphere. (C) Landmarking for corpus callosum thickness was performed from the midline (black arrows) and measurement locations are shown as red arrows. There was a trend toward a reduction in oligodendrocyte numbers in the corpus callosum in KO (D) but not in WT (E) injured animals. (F) Measurements for oligodendrocyte counts are delineated by red boxes. Representative immunofluorescence in the corpus callosum of KO Sham (G left), KO injured (G right), WT Sham (H left), and WT injured (H right) animals in the right and left hemispheres (blue = DAPI, green = Olig2). Scale bars = 50μm, 60x. Error bars = ±SD. * p<0.05, ** p<0.01.

The thickness of the corpus callosum was significantly decreased in KO injured animals (Fig 8A), but this was not observed in WT injured animals (Fig 8B). Measurement locations for the corpus callosum thickness are depicted in Fig 8C for the right and left hemispheres. There was a trend toward decreased numbers of oligodendrocytes in the corpus callosum in KO injured animals (Fig 8D and 8G right panel), but not in WT injured animals (Fig 8E and 8H right panel). Measurement locations for oligodendrocyte counts are depicted in Fig 8F for the right and left hemispheres.

#### Other structures (lateral ventricles, internal capsule)

**Fig 9:**
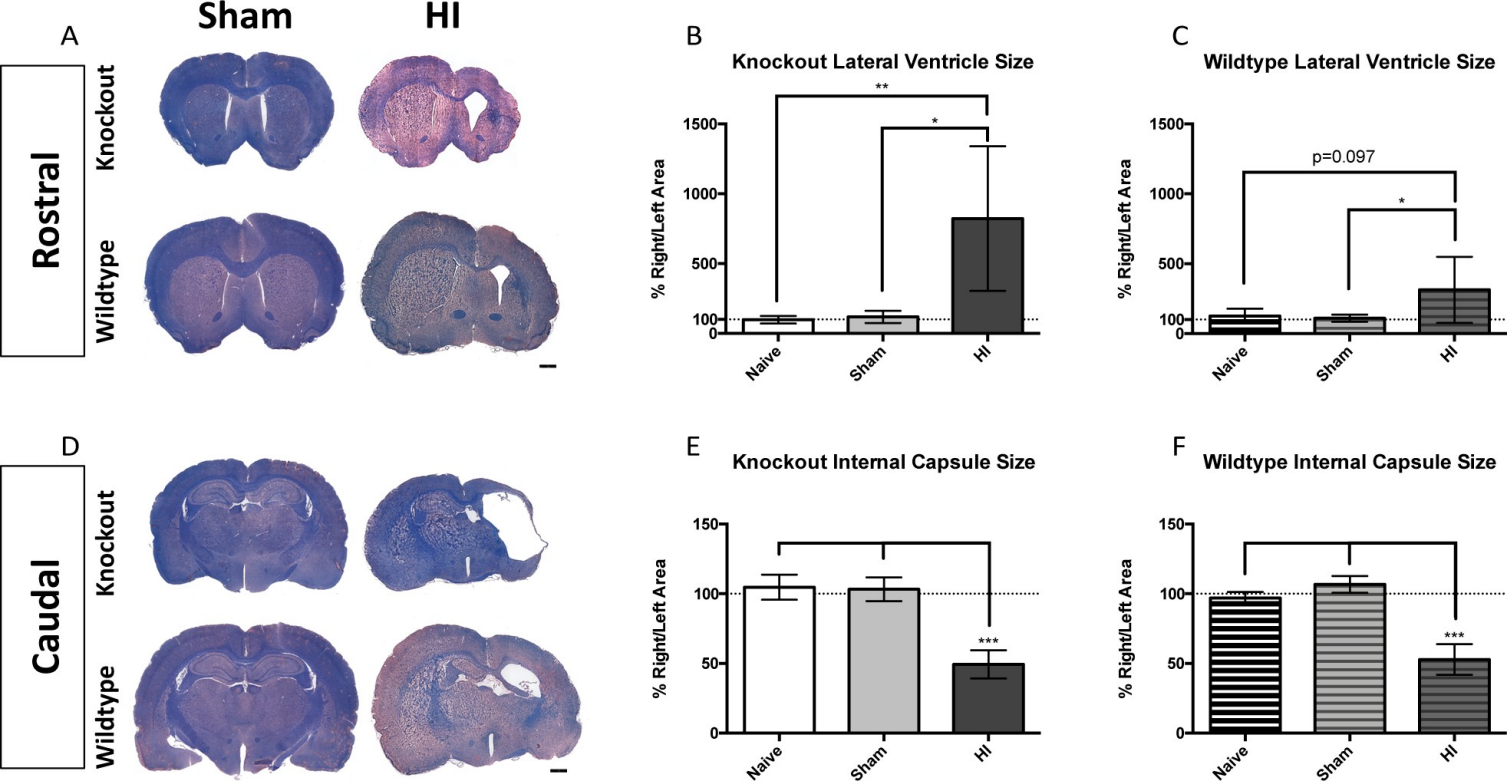
Lateral ventricle and internal capsule injuries. (A) Representative LFB/HE stained sections of Sham (left) and injured (right) animals of both genotypes at the rostral level. KO injured rats displayed significantly enlarged right ventricles (B), and there was a similar trend toward significance in WT rats (C). (D) Representative LFB/HE stained sections of Sham (left) and injured (right) animals of both genotypes at the caudal level. Both KO (E) and WT (F) injured animals exhibited a significant reduction in right internal capsule size. Scale bars = 1000μm, 5x. Error bars = ±SD. * p<0.05, ** p<0.01, ***p<0.001.

Representative rostral images of Sham and HI animals for both genotypes are displayed in Fig 9A. KO injured animals displayed significant ventriculomegaly in the right hemisphere (Fig 9B), while WT injured animals showed a trend toward ventriculomegaly in the right hemisphere (Fig 9C). Representative caudal images of Sham and HI animals for both genotypes are displayed in Fig 9D. The internal capsule size was reduced in both KO (Fig 9E), and WT (Fig 9F) injured animals.

### *Prkdc* knockout rats and wildtype rats are differentially affected by HI injury (Table 1)

In this study, injury was compared across KO and WT animals in order to investigate any differences in injury generation that were due to differences in genotype. Interestingly, several outcome measures revealed that KO rats are more susceptible to certain aspects of the injury than WT rats. The thickness of S1 was significantly more reduced in KO injured animals than in WT counterparts, and this was accompanied by a significant reduction in neurons in this region. There was a trend toward a difference in the thickness of M1 between KO and WT injured animals, but not in M1 neuronal counts. In the hippocampus, there was a trend toward a difference in area loss across genotypes, however, there were no differences between KO and WT injured animals in CA1 and CA3 neuronal loss. With regard to white matter, there was no significant difference in thickness of the corpus callosum between KO and WT injured animals, however, there was significantly more oligodendrocyte loss in the corpus callosum of KO injured animals. There were no differences between KO and WT injured animals in area of the internal capsule. Lateral ventricle size was significantly increased in KO injured animals when compared to WT injured animals. Finally, astrogliosis was significantly elevated in S1 and the CPu in KO injured animals, but not in the thalamus, and there were no differences in the extent of microgliosis in any brain region across genotypes.

**Table 1:** Effect of genotype on HI Injury. All Sham animals showed no differences in all measured parameters across genotypes. KO injured animals displayed increased severity of injury in the S1, corpus callosum, ventricles, as well as significantly elevated astrogliosis in S1 and the CPu when compared to WT injured animals. Adjusted p-values for the KO injured and WT injured comparison are shown. Those highlighted in yellow were significantly different across the two genotypes.

**Cortex**	
S1 Thickness	**p<0.0001**
M1 Thickness	p = 0.078
S1 NeuN Counts	**p = 0.0025**
M1 NeuN Counts	p = 0.699
**Hippocampus**	
Area	p = 0.071
CA1 NeuN Counts	p>0.999
CA3 NeuN Counts	p = 0.102
**White Matter**	
Corpus Callosum Thickness	p = 0.150
Corpus Callosum Olig2 Counts	**p = 0.017**
Internal Capsule Area	p = 0.401
**Ventricles & Gliosis**	
Ventriculomegaly	**p = 0.006**
S1% GFAP	**p = 0.036**
S1% Iba-1	**p = 0.412**
Cpu % GFAP	**p = 0.021**
Cpu % Iba-1	p = 0.421
Thalamus % GFAP	p = 0.619
Thalamus % Iba-1	p = 0.144

### Human iPSC-derived NPC survival is enhanced in *Prkdc* knockout rats

**Fig 10:**
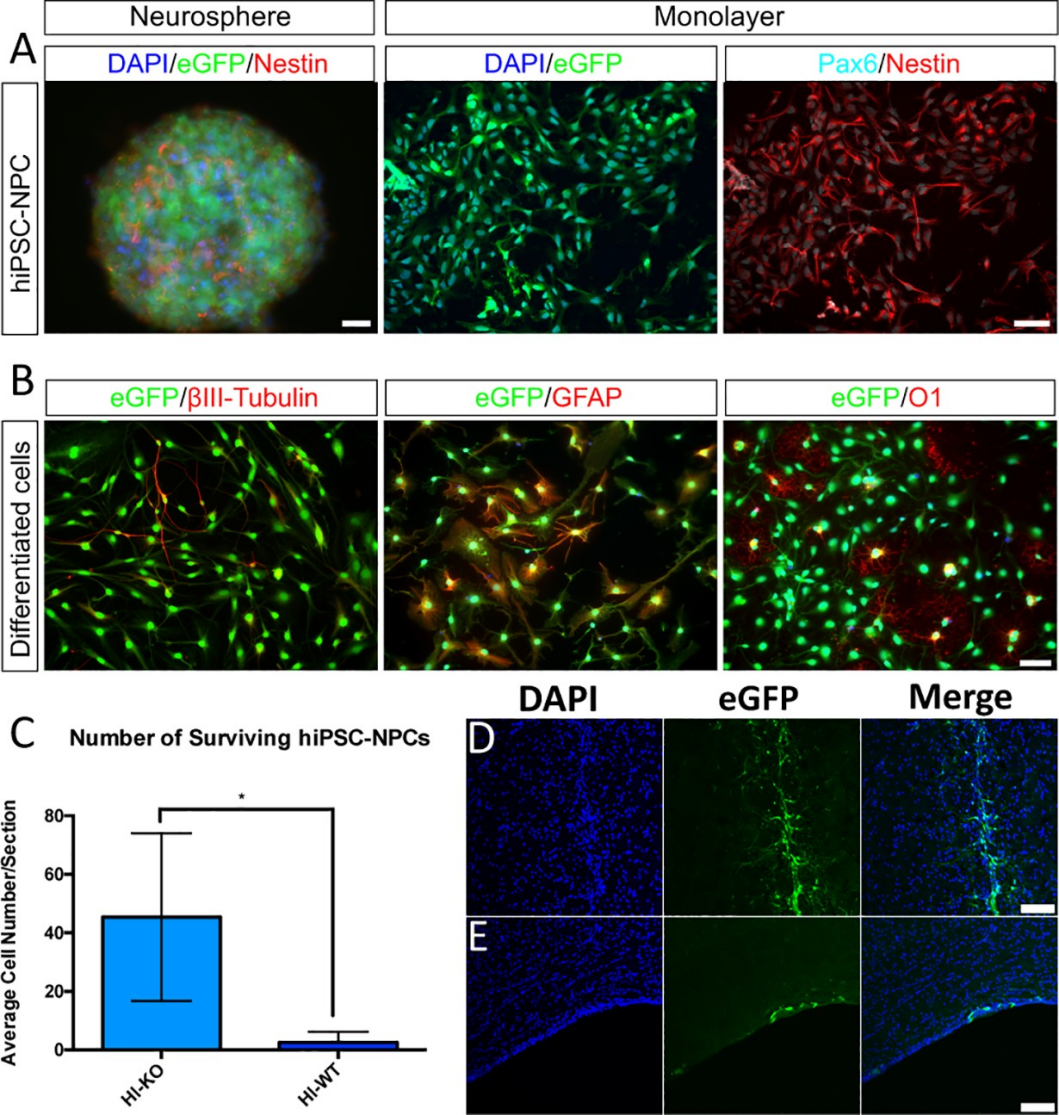
Increased survival of hiPSC-NPCs in KO injured animals. hiPSC-NPCs demonstrated the ability to form neurospheres (A left), express eGFP (A middle), and express the neural progenitor markers Nestin and Pax6 (A right). hiPSC-NPCs cultured under differentiation conditions expressed markers for neurons (B left), astrocytes (B middle), and oligodendrocytes (B right). Transplantation of these cells into the CPu of HI-injured animals resulted in significantly elevated cell survival in KO animals over WT animals at 10 weeks post-transplant (C). Representative images of eGFP-positive hiPSC-NPCs in KO injured (D) and WT injured (E) animals (blue = DAPI, green = eGFP). Scale bars A-B = 20μm, 20x. Scale bars D-E = 100μm, 20x. Error bars = ±SD. * p<0.05.

A stable hiPSC-NPC line expressing eGFP was generated using the *piggyBac* transposon system. The Neurosphere Assay demonstrated the ability of hiPSC-NPCs to generate neurospheres (Fig 10A left panel). They expressed the neural progenitor markers Nestin and Pax6 (Fig 10A right panel). We further confirmed the identity of the hiPSC-NPCs *in vitro* by evaluating their capacity to differentiate into all three neuroglial lineages. hiPSC-NPCs were able to differentiate into neurons (βIII-Tubulin^+^), astrocytes (GFAP^+^) and oligodendrocytes (O1^+^) (Fig 10B). At P9, hiPSC-NPCs were transplanted into the CPu of HI injured pups of both KO and WT genotypes. At 10 weeks post-transplant, the number of surviving eGFP^+^ cells that co-localized with DAPI was significantly higher in KO animals than in WT animals (Fig 10C). Representative images for KO (Fig 10D) and WT (Fig 10E) animals illustrate this difference.

## Discussion

This study sought to characterize a hemiplegic model of neonatal HI brain injury in SCID rats for the purpose of creating a platform to test human-derived NPCs as a treatment for neonatal stroke and CP. It has been well-documented that xenograft rejection is a major challenge to studying human-derived stem cells in preclinical animal models [23,26,32,33]. In addition, strain and species have been shown to significantly impact the generation of a neonatal HI brain injury [27]. Thus, this study is an essential first step to facilitate the translation of neural stem cell therapies to the clinic. Indeed, our findings indicate that a neonatal HI brain injury can be reliably reproduced in SCID rats. Despite missing both T and B cells, *Prkdc* KO rats display similar sensorimotor deficits to WT rats, though interestingly, demonstrate more severe brain damage in some regions of the brain at 10 weeks following injury. When designing the surgical parameters, we opted for less severe hypoxic conditions (75 minutes) than are often reported in the literature (>90 minutes) with the intent of minimizing the incidence of hemispheric liquefaction. This could explain the seemingly milder cortical injury, oligodendrocyte loss, and grey matter astrogliosis in WT animals [34].

While the aim of this study was not to dissect the impact of the SCID phenotype on neonatal HI brain injury, it is quite interesting that this condition seemed to exacerbate various aspects of the injury, but that the elevated tissue injury in KO animals did not correlate with worsened sensorimotor behavioural outcomes. A follow-up study would benefit from the use of other sensorimotor tests. Although the notion of which tests are “best” for use in stroke studies is widely debated [35], and some may be difficult to administer in pups as young as P21, additional testing could potentially help to dissect out any subtle functional differences between KO and WT injured animals.

Many components of the immune system have been implicated in the destructive pro-inflammatory processes that occur in the primary, secondary, and tertiary phases of neonatal HI brain injury [10,36,37], but literature on the role of T and B cells in this injury is minimal and conflicting [36,37]. We postulated that the increased severity in KO animals might be due to the absence of lymphocytes, and that this had a significant effect on the interplay among different components of the immune system, perhaps shifting the innate immune system to a more pro-inflammatory state and/or reducing the effects of anti-inflammatory cell populations [32,38]. Therefore, we sought to investigate whether or not microglia—the key immune-mediators in the CNS—played an exacerbated role in the KO animals. Microgliosis was clearly present in both KO and WT injured animals, with no difference across genotypes. This suggests that other components of the innate immune system may be functioning abnormally in response to the lack of T and B cells. Indeed, a recent study from Herz et al. (2018) provides evidence that pharmacologic blockade of T cells results in increased infiltration of innate immune components such as natural killer cells, neutrophils, dendritic cells, and macrophages into the brain, along with decreased infiltration of immunomodulatory T regulatory cells [38]. This study, combined with the results presented here, suggests that elements of the adaptive immune system play neuroprotective roles in the context of neonatal brain injury. Future investigation of CNS-infiltrating and peripheral leukocytes will help to shed light on the inflammatory aspects of neonatal HI. An alternative hypothesis is that the *Prkdc* gene plays a unique role in the brain, and when its protein product, DNA-dependent protein kinase catalytic subunit (DNA-PKc), is non-functional, this results in altered neurodevelopment and/or response to injury. There is indeed some evidence for the importance of DNA-PKc activity in repairing double-strand DNA breaks during neurogenesis, and *Prkdc* mutations in humans have been cited as causing some neurologic abnormalities [39,40], however the effects on the brain in the present injury model are unclear. A possible alternative to using *Prkdc* KO rats could be to use *RAG2* KO rats in the future, which also lack T and B cells. However, the improved utility of *RAG2* KO rats is merely speculative at this point, and the *Prkdc* mutation is much more widely studied. Regardless of the mechanisms behind the increased severity of injury in SCID rats, this research provides evidence that a neonatal HI brain injury is highly reproducible in *Prkdc* KO rats, establishing an excellent platform for preclinical testing of human-derived NPCs as a treatment for neonatal stroke and CP. Exacerbated damage in these SCID animals allows for a wider window through which to see the effects of human-derived NPC transplantation on recovery, and survival of transplanted cells in these rats was indeed improved compared to WT rats (discussed below).

Although the Rice-Vannucci model cannot recapitulate all aspects of the human condition, it is reliable and has been used extensively since its inception in 1981. It has allowed for the development of pivotal neuroprotective treatments, including hypothermia for at-risk term neonates [8,41]. Evaluation of sensorimotor deficits was a primary goal in this study, and the cylinder test revealed marked functional impairment in injured animals. The pasta test only showed minimal deficits, suggesting it may not be a useful outcome measure for this injury model. While we had initially also included two other sensorimotor tests (Noldus Catwalk and Ladderwalk), they were abandoned early in the study due to inconsistencies within the present injury model and high variance. Future studies would benefit from cognitive or social behavioural tests, in addition to the cylinder test.

Loss of neurons in the cortical S1 and hippocampal CA1, along with substantial gliosis in grey matter regions, provides numerous potential sites for targeting transplantation and assessing brain repair. A decrease in thickness and loss of oligodendrocytes in the corpus callosum also points to some effect of this injury on white matter, despite the P7 model mainly targeting vulnerable grey matter regions [5,42]. In this study, we chose to target the CPu as the site for transplantation. The CPu consistently displayed injury yet retained enough tissue integrity such that future studies looking to transplant at time-points beyond P9 would have viable tissue into which to inject. In addition, since our future goals will be to assess the effects of transplanted cells on motor function, the CPu seems to be a reasonable target.

Not only does this application of the classic Rice-Vannucci model reliably lead to injury in SCID rats, engraftment of hiPSC-NPCs was significantly higher in KO animals than in WT animals. In fact, there were almost no surviving cells in the WT animals, while cells survived in the KO animals until the 10-week post-transplant endpoint.

Previous research has not provided strong evidence for the long-term survival of human-derived NPCs in immune-competent rodent models of neonatal HI, if assessed at all [19–22]. While immune-suppression is sometimes considered as an alternative to using SCID rodents, there are concerns regarding the efficacy as well as the side effects that immune-suppressive compounds may have on the function of transplanted NPCs [23]. With that said, the lack of T and B cells in the *Prkdc* KO rats could also have an impact on the interplay between the transplanted cells and the brain microenvironment, and this is one of the major caveats to xenograft injury models. With this in mind, we did attempt to investigate if immune suppression in WT pups would be a reasonable alternative to immune-deficiency. However, immune-suppressed WT pups exhibited signs of malnourishment and an 80% (8/10 pups) mortality rate after 2 weeks of daily administration. Drug administration was discontinued on the surviving 2 pups and the approach was deemed impractical for use in this neonatal injury model. There are indeed several other SCID strains of rodents available that could have been used as alternatives to the *Prkdc* KOs. Athymic nude rats, which only lack mature T cells, are often used in transplant studies. While they have proven useful in this context, research has demonstrated the development of T-like cells with increasing age and rejection mediated by elevated natural killer cells [23,43]. In addition, these rats can still exhibit T-independent B cell reactions, which may partially contribute to acute transplant rejection [44]. Rats were selected over SCID mice for their more severe immunodeficient phenotype and lack of “leakiness” [25], ease of handling during behavioural testing, and that their larger size makes neonatal surgeries easier to perform.

Here, we present stark evidence that a SCID model of neonatal HI is essential for advancing translational studies of the therapeutic efficacy of human-NPC transplantation into the brain for neonatal stroke and CP. While the immune-deficient phenotype of SCID animals does not translate to the majority of human cases, and is thus a caveat to this model, immune rejection is a significant barrier to xenotransplantation of human-NPCs. This makes the use of SCID animals a highly relevant platform for preventing xenograft rejection and allowing for the long-term evaluation of this therapy in preclinical models. In this study, we chose to use iPSC-derived cells due to their clinical relevance and high translational potential. In the clinic, iPSC-derived cells can be taken from the patient themselves, thereby minimizing the risk of immune-rejection upon transplantation [28]. This makes the use of iPSC-NPCs much more feasible than using allogeneic NPCs. While the safety and efficacy of these hiPSC-NPCs for improving both functional and neuroanatomical recovery after HI-injury has yet to be assessed, this body of work provides evidence of the model’s necessity for future discordant xenograft studies.

## Conclusion

In this study we have shown that SCID rats can be used to generate a robust model of neonatal HI brain injury. Injuries at multiple locations in the brain provide various potential regions to target using human-derived NPCs. Transplantation and survival of hiPSC-NPCs in the HI-injured brain was successful in SCID animals, but not in immune-competent animals. Future research will be able to reliably utilize this xenograft model to test the safety and efficacy of human regenerative neural stem cell therapies by using a combination of sensorimotor and neurocognitive behavioural outcome measures, as well as by assessing the mechanisms through which hiPSC-NPCs function in the rodent HI brain.

## Supporting information

S1 Table Sample sizes. Sample sizes for each group are listed for each outcome measure used in the study.
(DOCX)

S1 Dataset (XLSX)

## Abbreviations

ANOVA: analysis of variance
ATP: adenosine tri-phosphate
BSA: bovine serum albumin
CNS: central nervous system
CP: cerebral palsy
CPu: caudate putamen
DAPI: 4’,6-diamidino-2-phenylindole
DMEM: Dulbecco’s modified eagle medium
DNA-PKc: DNA-dependent protein kinase catalytic subunit
EGF: epidermal growth factor
eGFP: enhanced green fluorescent protein
FBS: fetal bovine serum
FGF: fibroblast growth factor
GFAP: glial fibrillary acidic protein
HE: hematoxylin & eosin
HI: hypoxia ischemia
HT: heterozygote
hiPSC: human induced pluripotent stem cell
kDa: kilodalton
KO: knockout
iPSC: pluripotent stem cell
LFB: luxol fast blue
M1: primary motor cortex
NEM: neural expansion media
NeuN: neuronal nuclei
NMDA: N-methyl-D-aspartate
NIM: neural induction media
NPC: neural precursor cell
OCT: optimal cutting temperature (compound)
Pax6: paired box protein 6
PBS: phosphate buffered saline
PFA: paraformaldehyde
PLL: poly-L-lysine
ROI: region of interest
SCID: severe combined immunodeficient
SD: standard deviation
S1: primary somatosensory cortex
TGFβ: transforming growth factor beta
WT: wildtype
