# Supplementary material for: Severe-combined immunodeficient rats can be used to generate a model of perinatal hypoxic-ischemic brain injury to facilitate studies of engrafted human neural stem cells
Source: PLoS One. 2018 Nov 28;13(11):e0208105. doi: 10.1371/journal.pone.0208105 (PMC6261629; doi:10.1371/journal.pone.0208105)
Supplement: S1 Table — Sample sizes for each group are listed for each outcome measure used in the study. (DOCX) [file pone.0208105.s001.docx]

**S1 Table. Sample Sizes.** Sample sizes for each group are listed for each outcome measure used in the study.

| **Outcome Measure** | **Knockout** | | | **Wildtype** | | |
| --- | --- | --- | --- | --- | --- | --- |
|  | **Naïve** | **Sham** | **HI** | **Naïve** | **Sham** | **HI** |
| **Body Weight** | 12 | N/A | | 10 | N/A | |
| **Spleen Size** | 11 |  |  | 8 |  |  |
| **Cylinder Test** | 10 | 11 | 14 | 9 | 12 | 13 |
| **Pasta Test** | 12 | 12 | 18 | 9 | 14 | 14 |
| **S1 Size** | 6 | 7 | 10 | 7 | 7 | 11 |
| **M1 Size** | 6 | 6 | 11 | 7 | 7 | 11 |
| **S1 NeuN Counts** | 6 | 7 | 9 | 6 | 7 | 8 |
| **M1 NeuN Counts** | 6 | 7 | 9 | 6 | 7 | 8 |
| **Hippcampal Size** | 7 | 7 | 11 | 7 | 7 | 11 |
| **CA1 NeuN Counts** | 7 | 7 | 8 | 7 | 7 | 7 |
| **CA3 NeuN Counts** | 5 | 7 | 8 | 7 | 5 | 8 |
| **S1 GFAP** | 4 | 6 | 7 | 6 | 6 | 8 |
| **Cpu GFAP** | 4 | 6 | 8 | 6 | 6 | 8 |
| **Thalamus GFAP** | 6 | 6 | 8 | 6 | 6 | 9 |
| **Iba-1 (All)** | 6 | 6 | 6 | 6 | 6 | 6 |
| **Corpus Callosum Thickness** | 6 | 7 | 10 | 7 | 6 | 11 |
| **Corpus Callosum Olig2 Counts** | 5 | 6 | 8 | 7 | 7 | 9 |
| **Lateral Ventricle Size** | 7 | 7 | 11 | 7 | 7 | 11 |
| **Internal Capsule Size** | 7 | 7 | 11 | 7 | 7 | 11 |
| **hiPSC-NPC Transplantation** | N/A | | 6 | N/A | | 4 |
